# Everybody's Page

**Published:** 1890-12-27

**Authors:** 


### Everybody's Page.

"Well, Pat, I'm told you were nearly killed by the cold in America." "'Deed, an' I was thin. Shure, 'twas often 40 degrees below Nero, wheriver he may be."

---

A Welsh jury, taking a fatherly interest in inebriates, has held up as a warning to drunkards during the coming Christmas festivities the death of a discharged soldier who accomplished in an hour-and-a-half the remarkable feat of drinking a pint of whisky and quantities of rum. The quantities of rum are not stated, but the mixture apart from the quantity ought to have been enough to cause a fatal result.

---

The popular supposition embodied in Byron's famous lines, and recorded as a fact of Mary Queen of Scots and Marie Antoinette, that people's hair can and does and has become white suddenly, has frequently been disputed by pathologists whose theories, however, Dr. Prentiss has set to work in his recent book to disprove, conclusively showing the falseness of the doctrine that the hair has no nerve connection with the general system, and so cannot be affected either gradually or suddenly by violent emotions, such as fright, grief, joy, or any unusual shock.

---

The need of sufficient sleep for young people, and especially children, is only too frequently ignored. The result of late hours is to be seen only too painfully and clearly in the case of the former during a London season. They, however, have the remedy in their own hands; but with children it is different, and too many mothers seem blind to the value, to children under five years of age, of the two hours' morning sleep. This is just as legitimate a portion of a child's sleep as its night's rest, and to rob a child of it has a most pernicious influence on the child's healthy development. Healthy children will sleep the clock round at night, and should never be roused from a sound sleep.

---

It is reported of a certain celebrated doctor that a learned professor, who was a patient of his, caused him no small though momentary astonishment at the suddenness with which he acted upon his medical mentor's advice. The doctor, having written a prescription for a tonic, impressed upon his patient the absolute necessity of recreation, in the efficacy of which in this individual case he believed in more than in drugs. "Here is your prescription," he said, "but what you want is a good hearty laugh." No sooner had the professor glanced at the prescription than he began to laugh immoderately. The surprised doctor exclaimed, "What *are* you laughing at?" "Why, at your Latin," replied the professor.

Dr. Brown Sequard's fluid is once more to the fore, this time as a competitor with Dr. Koch's lymph. A nostrum which can transform a decrepit Methuselah into a youthful Hercules would naturally rise superior to such simple affairs as tuberculosis and lupus.

---

It appears to be Professor Goldwin Smith's opinion that no boy should be sent to college—and we presume he includes school—who does not show a decided inclination for study. We have hitherto looked upon boys, apart from prigs, who have a decided inclination for study, as similar to snakes in Iceland.

---

The effects of such fogs as have been experienced in London during the present month are very marked on plant life. Aquatic and soft or tender-leaved plants appear to suffer more than others, especially from the deleterious agents which town fogs contain, but, as Professor Bentley points out, tropical plants, the natural habitat of which is one exposed to sunshine, feel in a particular degree the destructive action of fog owing to the consequent want of light.

---

If Mons. Armand Gautier, the distinguished member of the Hygienic Council of the French capital, is a true prophet, the Paris of the future will be a very different place from the gay and bright city it still is. Foggy and smoky London has become, and not altogether unjustly, a by-word amongst the fortunate inhabitants of sunnier climes, and Mons. Gautier threatens his fellow-citizens with a similar fate, and the cause of the impending calamity is, as he thinks, the numerous steam engines now being used for the production of the electric light.

---

The difference between Hindoo and Mohammedan superstitions was illustrated in a curious manner recently in India. To the Hindoos every supernatural being is a god, but to the Mohammedan only a ghost or hobgoblin; and this difference of creed was exemplified in the case of a piece of land which was supposed to be haunted. The Hindoos having tried in vain to propitiate the goddess whom they supposed to be in residence, the Mohammedans proceeded, with success, to reclaim the land by defying the ghost. This they did by killing a cow at the tree which the Hindoos had set apart for the divine residence, hanging some of the flesh in the branches, and smearing the tree with blood. Such an outrage was more than the Hindoos could bear; they set the law in motion, and several of the Mohammedans were imprisoned for "outraging religious susceptibilities." Whether the Hindoos still look upon the land as haunted, or consider that having thus avenged the goddess they have propitiated her, we are not aware.

